# Pathogenic functions of host microbiota

**DOI:** 10.1186/s40168-018-0542-0

**Published:** 2018-09-28

**Authors:** Silke Rath, Tatjana Rud, André Karch, Dietmar Helmut Pieper, Marius Vital

**Affiliations:** 1 Microbial Interactions and Processes Research Group, Helmholtz Centre for Infection Research, Braunschweig, Germany; 2 Epidemiological and Statistical Methods Research Group, Helmholtz Centre for Infection Research, Braunschweig, Germany

**Keywords:** Pathogen, Gut microbiota, Function, Ecology, Systems biology, Diagnostics, Risk assessment, TMA, Bile, Hydrogen sulfide

## Abstract

**Background:**

It is becoming evident that certain features of human microbiota, encoded by distinct autochthonous taxa, promote disease. As a result, borders between the so-called opportunistic pathogens, pathobionts, and commensals are increasingly blurred, and specific targets for manipulating microbiota to improve host health are becoming elusive.

**Results:**

In this study, we focus on the functions of host bacterial communities that have the potential to cause disease, proposing the term “pathogenic function (pathofunction)”. The concept is presented via three distinct examples, namely, the formation of (i) trimethylamine, (ii) secondary bile acids, and (iii) hydrogen sulfide, which represent metabolites of the gut microbiota linked to the development of non-communicable diseases. Using publicly available metagenomic and metatranscriptomic data (*n* = 2975), we quantified those pathofunctions in health and disease and exposed the key players. Pathofunctions were ubiquitously present with increased abundances in patient groups. Overall, the three pathofunctions were detected at low mean concentrations (< 1% of total bacteria carried respective genes) and encompassed various taxa, including uncultured members.

**Conclusions:**

We outline how this function-centric approach, where all members of a community exhibiting a particular pathofunction are redundant, can contribute to risk assessment and the development of precision treatment directing gut microbiota to increase host health.

**Electronic supplementary material:**

The online version of this article (10.1186/s40168-018-0542-0) contains supplementary material, which is available to authorized users.

## Background

Pathogens are classified as bacteria capable of causing host damage via specific virulence factors that encompass production of toxins, features allowing attachment to and invasion of epithelial cells and components essential for their viability [1]. Definitions of pathogens and associated virulence factors have been continuously adjusted over the last decades proposing additional aspects to be considered for pathogenicity such as host physiology, where certain bacteria are only able to cause disease in immunocompromised subjects [1]. Additionally, the term pathobiont was introduced to describe commensal, harmless bacteria that can turn hostile under specific circumstances [2]. Methodological advancements in the last decade enabled detailed insights into whole bacterial assemblages and expanded investigations to the community level introducing the prominent term “dysbiosis” that describes altered community structures of host microbiota associated with disease [3]. Recently, the “germ-organ theory” was introduced suggesting oxygen to be the main driver of dysbiosis that is accompanied by a bloom of facultative anaerobic *Proteobacteria* [4]. As a result, gut homeostasis is disrupted leading to disease due to dysfunction of the microbial organ. The “pathobiome” concept represents another community-wide approach and encompasses all pathogenic agents integrated within their biotic environment [5]. It is organism-centric describing the collection of potentially pathogenic microorganisms in a given community. In hand with those broader concepts and terms describing bacteria (and whole communities) damaging the host, borders between the so-called commensals, pathobionts, and opportunistic pathogens are increasingly blurred, and specific community-wide targets for manipulating microbiota to improve host health are becoming elusive.

In this study, we introduce the term “pathogenic function (pathofunction)” representing specific features of host bacterial communities that have the potential to cause non-communicable disease. Pathofunctions comprise various modes of action such as the production of harmful metabolites, extracellular enzymes, or immunostimulatory surface structures (Table 1). Host damage is a direct result of pathofunction activity or due to the initiation of harmful downstream processes like immune system dysbalances and usually requires longer-term exposure and/or excessive concentrations for causing disease. Importantly, the concept focuses on the functions that are shared by various, taxonomically distinct organisms, which distinguish pathofunctions from traditional virulence factors that have a functional perspective too, yet are restrictedly used for characterizing particular bacteria/strains as pathogens. Furthermore, pathofunctions do not comprise infection where the disease is caused by intruding bacteria encoding functions that are not autochthonous to the host environment. We do not consider viability features of pathofunction-carrying bacteria such as components promoting their growth or facilitating immune system evasion as pathofunctions if they are not directly involved in the disease development. In summary, the pathofunction concept involves two key aspects: it is (i) function-centric and (ii) encompasses whole commensal communities, where all members exhibiting a particular pathofunction are redundant. Its potential contribution to risk assessment and the development of intervention strategies to increase host health is discussed.

**Table 1:** Selected (putative) pathofunctions of gut microbiota and associated diseases

Pathofunction	Mode	Associated disease	References
TMA(O)	Metabolite	CVD, T2D, kidney disease	[8, 9]
LCA/DCA	Metabolite	CRC, liver cancer	[13]
Hydrogen sulfide	Metabolite	IBD, pouchitis, CRC	[19, 20]
Indole/phenol/p-cresol	Metabolite	IBD, CVD, renal failure	[54]
*N*-Nitrosamine	Metabolite	Stomach cancer	[55]
Ammonia	Metabolite	Several conditions (e.g., hepatic encephalopathy)	[56]
Branched-chain amino acids	Metabolite	Obesity-associated insulin resistance	[37]
4-Ethylphenylsulfate	Metabolite	Neurodevelopmental disorders	[57]
Uric acid	Metabolite	Gout	[58]
Bacterial proteases	Enzyme	IBD	[59]

For displayed metabolites, pathofunctions represent enzymes catalyzing their formation

*CRC* colorectal cancer, *CVD* cardiovascular disease, *IBD* inflammatory bowel disease, *LCA/DCA* lithocholic/deoxycholic acid, *T2D* type 2 diabetes, *TMA(O)* trimethylamine (*N*-oxide)

## Results and discussion

In this study, we investigated three distinct pathofunctions, namely, the microbial formation of (i) trimethylamine (TMA), (ii) secondary bile acids deoxycholic and lithocholic acid (DCA/LCA), and (iii) hydrogensulfide (H_2_S), in order to expose various characteristics of pathofunctions and to outline strategies/challenges for diagnostics and treatment.

TMA is produced from dietary quaternary amines mainly via three distinct enzymatic routes with betaine, choline, and carnitine as substrates. Various distinct taxa are reported to encode respective enzymes [6, 7] highlighting that pathofunctions can exhibit both biochemical (different pathways) and taxonomic redundancies. Host hepatic flavin monooxygenases (FMO) subsequently oxidize absorbed TMA to trimethylamine *N-*oxide (TMAO) that is associated with atherosclerosis and severe cardiovascular disease [8] as well as kidney disease [9]. It is postulated that TMAO promotes disease through the formation of foam cells (lipid-laden macrophages), a diminishing of the reverse cholesterol transport from the atherosclerotic plaque [10], and enhances platelet reactivity [11]. Recent gene-targeted studies ubiquitously detected potential TMA-producing bacteria, primarily belonging to *Clostridiales* and *Enterobacteriaceae*, in the gut of human, where they constitute, however, only a minor part of the total community (below 1% in most samples) with key players yet to be isolated [7, 12].

The secondary bile acids DCA and LCA are formed by gut bacteria via the multistep 7α-dehydroxylation from cholic acid and chenodeoxycholic acid, respectively. They promote cancer of the colon and the liver via various cytotoxic effects and immune system modulations [13, 14]. A few intestinal *Clostridiales* strains capable of 7α-dehydroxylation have been isolated, though data on their abundance in situ and major taxa involved are scarce. LCA and DCA are detected in most humans suggesting that respective bacteria are ubiquitously present [15].

Anaerobic respiration with sulfate, sulfite, or organosulfonates as terminal electron acceptors is widespread in various ecosystems. It is performed by the members of many distinct taxa from both *Eubacteria* and *Archaea* [16], where the dsrAB-type dissimilatory (bi)sulfite reductase forming sulfide from sulfite is the key enzyme. In the gut, bacteria acting on sulfate or organic sulfur-containing compounds including mucin, taurine, and amino acids are ubiquitously detected at low abundances [17]. *Desulfobacterales* and *Desulfovibrionales*, particularly *Desulfovibrio* and *Bilophila* (the latter does not reduce sulfate), are the key players using fermentation end products (e.g., short-chain fatty acids) and H_2_ as electron donors [18]. At excessive concentrations, H_2_S is a cytotoxic gas associated with inflammatory conditions of the gut epithelium such as ulcerative colitis and pouchitis [19] as well as colorectal cancer [20].

### Quantification and characterization of pathofunctions

For accurate diagnostics of any particular pathofunction, its entire pathogenic potential including all biochemical pathways and respective bacteria carrying the function (“carriers”) should be resolved rendering metagenomics as the method of choice. In this study, we screened publicly available metagenomic and metatranscriptomic datasets comprising conditions (and comorbidities) associated with the three pathofunctions introduced above in order to get detailed insights into their relation with disease. Datasets originated from three continents (Asia, Europe, North America) and encompassed cardiovascular disease (CVD: I [21], II [22]), type 1(2) diabetes (T1(2)D: III [23], IV [24], V [25]), obesity (VI [26]), colorectal cancer (CRC: VII [27], VIII [28], IX [29]), liver cirrhosis (X [30]), and inflammatory bowel disease (IBD: XI [31] and XII [32]), with respective healthy controls (Table 2). Only a little information on the three pathofunctions is available from original studies (Additional file 1). The following databases were used for screening. For TMA, sequences of choline-lyase (*cutC*), and its activator *cutD* as well as carnitine oxygenase/reductase (*cntA/B*) from Reference [7] were applied. Databases of genes encoding betaine reductase (*grdH*) forming TMA from betaine as well as of genes of the bile acid inducible (bai) operon (*baiA-I*) encoding enzymes catalyzing the 7α-dehydroxylation of cholic/chenodeoxycholic acid to DCA/LCA were established in this study (see the “Methods” section). For *dsrA/B* (H_2_S formation), the comprehensive, manually curated database provided by Müller et al. [16] was used. Enzymatic routes encompassing the conversion of sulfur-containing amino acids as well as endogenic H_2_S generation from the host were not considered here.

**Table 2:** Overview of individual datasets included in this study

Study	Reference	Short description	Continent
I	Jie et al. [21]	CVD (*n* = 218) vs. controls (*n* = 186)	A
II	Karlsson et al. [22]	CVD (*n* = 13) vs. controls (*n* = 12)	E
III	Qin et al. [23]	T2D (*n* = 182) vs. controls (*n* = 185)	A
IV	Forslund et al. [24]	T2D (*n* = 75), T1D (*n* = 31) vs. samples from VI	E
V	Karlsson et al. [25]	T2D (*n* = 43) vs. controls (*n* = 53)	E
VI	Le Chatelier et al. [26]	Obese (*n* = 161) vs. controls (*n* = 109)	E
VII	Feng et al. [27]	CRC (*n* = 46), LA (*n* = 47) vs. controls (*n* = 63)	A/E
VIII	Zeller et al. [28]	CRC (*n* = 91), LA (*n* = 15), SA (*n* = 27) vs. controls (*n* = 66)	E
IX	Vogtmann et al. [29]	CRC (*n* = 52) vs. controls (*n* = 52)	NA
X	Qin et al. [30]	Cirrhosis (*n* = 123) vs. controls (*n* = 114)	A
XI	Qin et al. [31]	UC (n = 21), CD (*n* = 4) vs. controls (*n* = 14)	E
XII	Schirmer et al. [32]	MTG: UC (*n* = 78), CD (*n* = 175) vs. controls (*n* = 55)	NA
		MTX: UC (*n* = 46), CD (*n* = 121) vs. controls (*n* = 11)	
XIII	Mehta et al. [53]	MTG and MTX of 78 subjects (4 time points)	NA

A/E—fecal matter derived from European subjects, whereas sample processing was performed in China

*CD* Crohn’s disease, *CRC* colorectal cancer, *CVD* cardiovascular disease, *LA* large adenoma, *SA* small adenoma, *T1(2)D* type 1(2) diabetes, *UC* ulcerative colitis, *MTG* metagenome, *MTX* metatranscriptome, *A* Asia, *E* Europe, *NA* North America

**Fig. 1:**
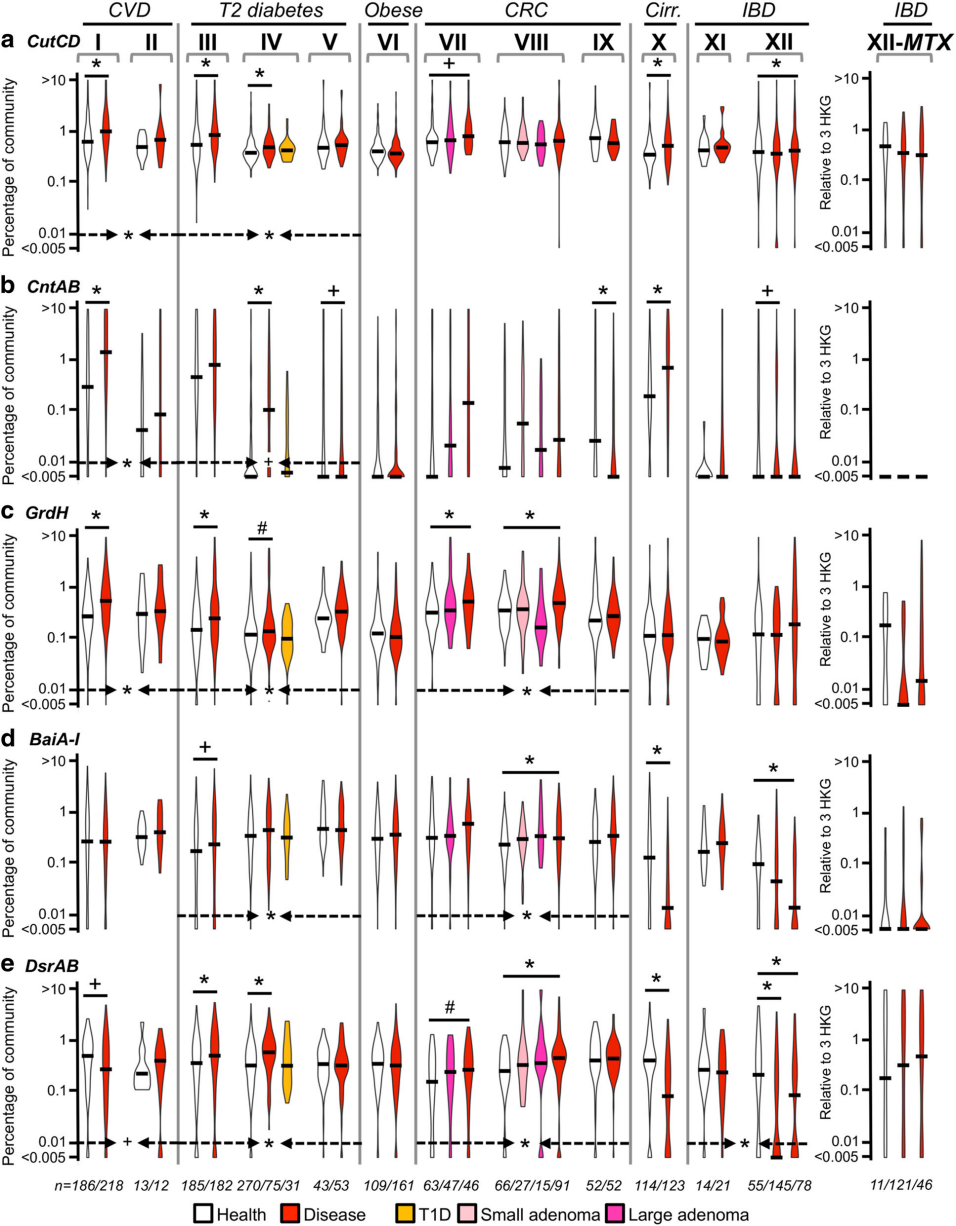
Association between pathofunction abundance and disease. Abundances of genes encoding three pathofunctions, namely, the formation of (i) trimethylamine (*cutCD* (**a**), *cntAB* (**b**), *grdH* (**c**)), (ii) the secondary bile acids lithocholic/deoxycholic acid (*baiA-I* (**d**)), and (iii) hydrogen sulfide (*dsrAB* (**e**)), were quantified in metagenomic data encompassing patients (red violin plots) suffering from cardiovascular disease (CVD: I, II), type 2 diabetes (T2 diabetes: III–V), obesity (Obese: VI), colorectal cancer (CRC: VII-IX), liver cirrhosis (Cirr: X), and inflammatory bowel disease (IBD: ulcerative colitis (UC) and Crohn’s disease (CD), XI, XII) and compared with healthy controls (white violin plots). In dataset XII results of CD patients are displayed following those from UC patients (on the right), whereas CD patients of dataset XI are not shown due to the low sample size (*n* = 4). For the key of colors of additional violin plots, see legend at the bottom of the figure. Pathofunction abundance refers to the percentage of total bacteria of a sample carrying respective genes, i.e., relative to the mean abundance of three single-copy housekeeping genes (HKG). Gene expression results (MTX) are displayed relative to the mean expression of those HKG. Black bars in violin plots represent median values. Significant differences (*p < 0.05) and trends (^+^*p* < 0.1 and ^#^*p* = 0.1) between patients and healthy controls are indicated (Student’s *t* test on log-transformed (log(*x* + 1)) data, whereas each disease results of generalized linear mixed-effects models using sample origin (dataset) as a random effect are indicated at the bottom of each plot within dashed arrows

Pathofunctions were detected in most samples of all datasets at similar mean abundances with ~ 0.1–1% of total bacteria carrying respective functions; only a few individuals displayed abundances > 1% (Fig. 1). The TMA-formation potential from carnitine was an exception with many samples lacking this function, while others displayed high abundances, especially those originating from Chinese individuals (I, III, X). CVD patients were enriched in genes encoding formation of TMA, where all three pathways were elevated in both datasets compared with healthy controls and displayed an area under the receiver-operating characteristic curve (AUC) of 0.71 for combined TMA data in regression analysis based on generalized linear mixed effect models (GLMM) (Additional file 2). Subjects suffering from type 2 diabetes (T2D) exhibited increased mean abundances of all three pathofunctions (III, IV; in dataset V patients showed similar levels as controls) with TMA exhibiting highest AUCs (Additional file 2). No significant alterations in type 1 diabetic (T1D) individuals from healthy controls were detected (IV) suggesting that a glycemic phenotype was not responsible for the observed increases in T2D patients. Obese subjects were unaffected, whereas abundances of all pathofunctions (*grdH*, *baiA-I*, and *dsrAB*) were significantly elevated in colorectal cancer (CRC) patients compared with controls (Fig. 1). Cirrhotic individuals had increased levels of TMA producers (*cutCD* and *cntAB*), yet other pathofunctions were even decreased compared with healthy controls. No differences in abundance of any pathofunction were detected in patients suffering from IBD in dataset XI, and *dsrAB* as well as *baiA-I* showed even decreased abundances based on metagenomic data derived from dataset XII. However, results based on gene expression indicated no significant differences between the groups. In conclusion, pathofunction abundances were increased in diseases where an etiological role has been previously proposed such as increased TMA-producing potentials in CVD patients and elevated levels of both *bai* and *dsr* genes in individuals suffering from CRC. Unexpectedly, the H_2_S formation potential was not increased in IBD (Fig. 1). Higher concentrations were additionally detected in distinct patient groups, most prominently the increased abundances of genes encoding for TMA formation in T2D subjects. Those findings are consistent with analytical measurements that revealed increased TMAO levels in such patients [33], yet its role in the development of disease is not clear.

**Fig. 2:**
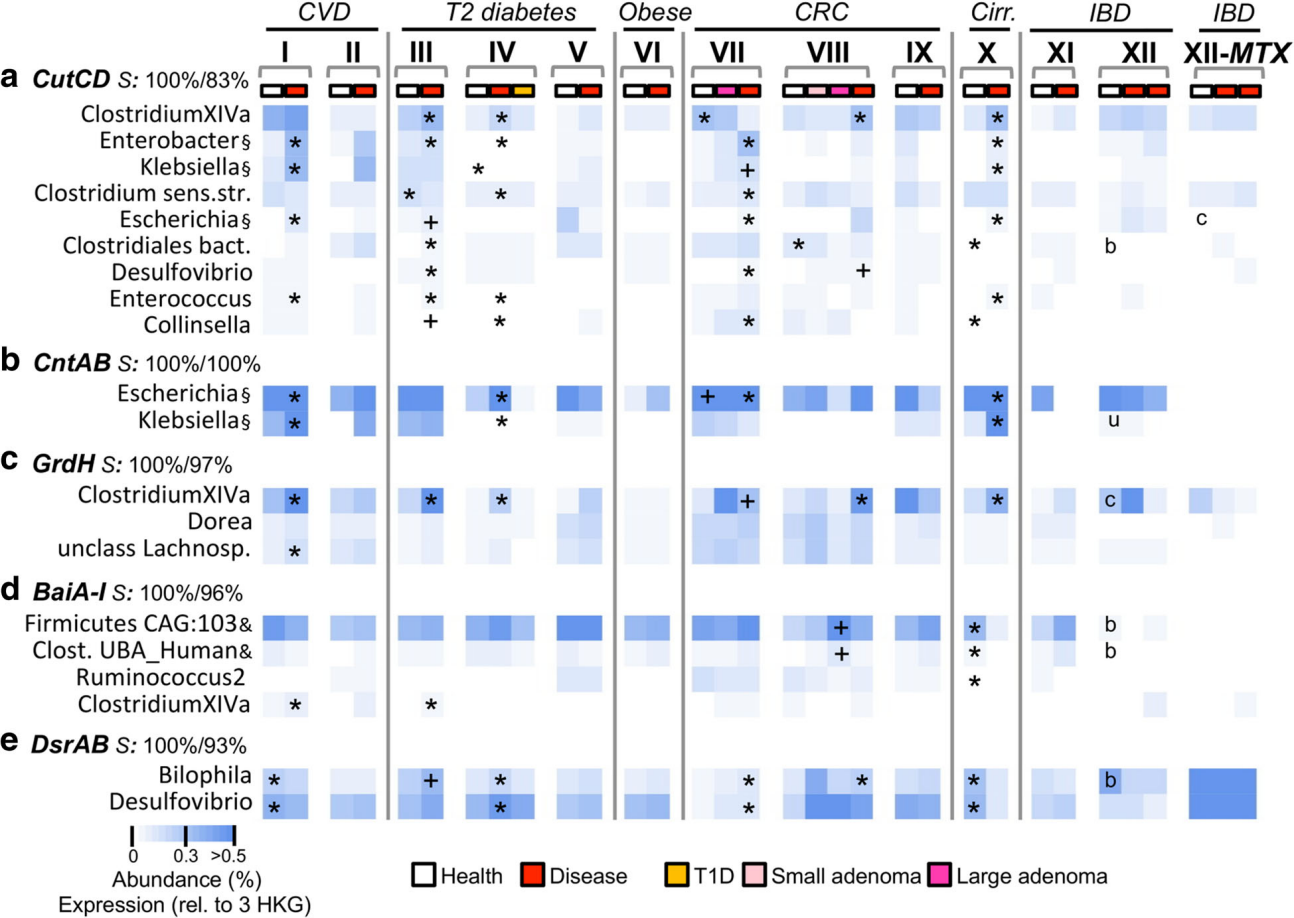
Abundance of individual bacteria carrying pathofunctions. Taxonomic affiliations of genes encoding three pathofunctions, namely, the formation of (i) trimethylamine (*cutCD* (**a**), *cntAB* (**b**), *grdH* (**c**)), (ii) the secondary bile acids lithocholic/deoxycholic acid (*baiA-I* (**d**)), and (iii) hydrogen sulfide (*dsrAB* (**e**)) were quantified in metagenomic data encompassing patients suffering from cardiovascular disease (CVD: I, II), type 2 diabetes (T2 diabetes: III–V), obesity (Obese: VI), colorectal cancer (CRC: VII-IX), liver cirrhosis (Cirr: X), and inflammatory bowel disease (IBD: ulcerative colitis (UC) and Crohn’s disease (CD), XI and XII) and compared with healthy controls. In dataset XII results of CD patients are displayed following those from UC patients, whereas CD patients of dataset XI are not shown due to the low sample size (*n* = 4). Taxa abundance refers to the percentage of total bacteria of a sample carrying respective genes, i.e., relative to the mean abundance of three single-copy housekeeping genes (HKG). For expression data (MTX) results are expressed relative to the mean expression of those HKG. Significant differences (*p < 0.05) and trends (^+^*p* < 0.1) between patients and healthy controls are shown (FDR-corrected Mann-Whitney *U* test). For CRC, “*” in healthy controls indicates significant difference (*p* < 0.05) from cancer patients, whereas in IBD datasets “u” and “c” refer to significantly different results (*p* < 0.05) in healthy controls compared with UC and CD, respectively, whereas “b” indicates the differences of both diseases from controls. The most abundant taxa that cumulatively represent 67% (**a**), 94% (**b**), 86% (**c**), 93% (**d**), and 81% (**e**) of total abundances of individual pathofunctions are displayed. Read similarities (S:%,) are indicated as median/first quartile protein similarities to top-hitting reference on top of each panel. §: affiliated with *Enterobacteriaceae*; &: affiliated with metagenomic species; Clostridium sens. str.: *Clostridium* sensu stricto; Clostridiales bact.: SAMEA3545284 (unclassified *Clostridiales*); Clost. UBA Human: *Clostridiales bacteria* UBA6412, UBA4701, and UBA5888; Firmicutes CAG:103: *Firmicutes bacterium* CAG:103; unclass Lachnosp.: unclassified *Lachnospiraceae*

Several genera were detected carrying individual pathofunctions, demonstrating functional redundancy of taxonomically distinct bacteria, particularly in the case of *cutCD* where members belonging to two distinct phyla *Proteobacteria* and *Firmicutes* were revealed as key members, as observed earlier [7, 34]. In line with previous work on genes encoding enzymes for TMA formation [7], individual samples contained taxonomically distinct bacteria carrying pathofunctions, though insights into their richness per subject remained rudimentary due to low sequencing depth. Resolutions gained from gene-targeted approaches such as investigations on *cutC*-exhibiting communities [7] are able to provide more detailed insights in carrier diversity, though enumeration can be difficult if many different pathways are involved and target sequences display high heterogeneity. Overall, reads from metagenomes were closely matching references displaying median protein similarities of 100% (Fig. 2) suggesting that most important taxa in situ are represented in our databases. However, many *cutCD*-linked reads showed lower values where 25% of reads displayed similarities ≤ 83%, and a majority of reads matching genes encoding 7α-dehydroxylation of bile acid (baiA-I) was associated with two metagenomic species (Additional file 3) indicating that key members carrying those pathofunctions are yet to be isolated.

*CutCD* genes previously found in various taxa were concurrently driving the abundance increase of the total pathway in patient groups (Fig. 2). Occasionally, disparate abundance alterations of taxa during disease were detected, such as *Clostridium* sensu stricto that, against the common trend, decreased in T2D (III) and cirrhotic (X) patients, underlining distinct ecology of individual *cutCD*-carrying bacteria. Main members of *cntAB* and *grdH* carriers, i.e., *Escherichia*/*Shigella*, *Klebsiella*, and *Clostridium* XIVa, respectively, were governing elevation of other TMA-forming pathways. Potential 7α-dehydroxylating taxa containing *baiA-I*, particularly the metagenomic species *Firmicutes bacterium CAG:103*, that recruited > 60% of *bai*-associated reads trended increased in CRC patients (Fig. 2, Additional file 3). *Bai* genes previously described in *Clostridium* XIVa displayed higher levels in CVD and T2D patients compared with healthy control groups. The main *dsrAB*-containing taxa, *Desulfovibirio* and *Bilophila*, showed similar behavior and governed total pathway alterations in patient groups.

**Fig. 3:**
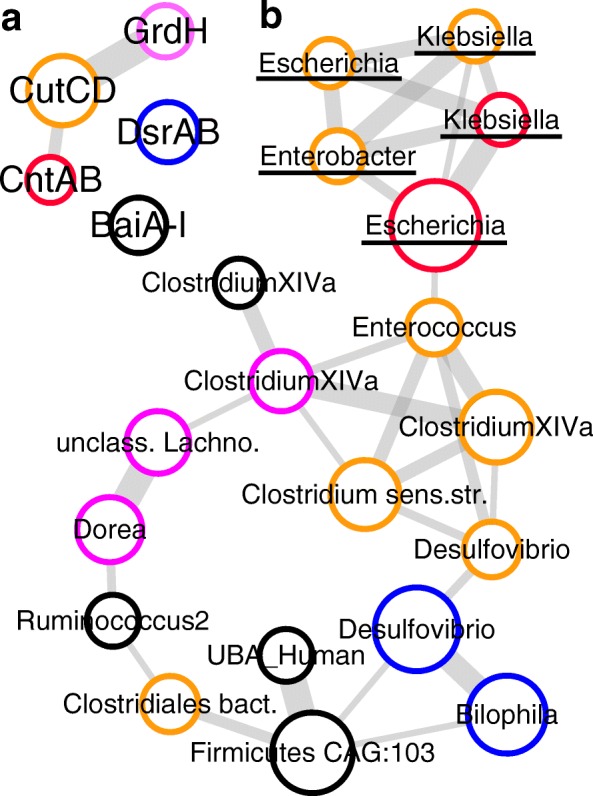
Co-occurrence analysis of individual pathofunctions. Analyses were performed on abundance data derived from 12 datasets (Table 2). Datasets IV and VI were merged as they derived from one source and have overlapping samples in the healthy control group. Only metagenomic results of datasets XII and XIII were considered. Edge-width represents correlation strength defined as the number of datasets displaying a correlation (*p* and *q* < 0.05 and Spearman’s rho > 0.35; a minimum of three correlations was required for connecting individual nodes), and node sizes reflect median abundances. Abundances of genes encoding the formation of (i) trimethylamine (*cutCD* (orange), *cntAB* (red), *grdH* (pink)), (ii) the secondary bile acids lithocholic/deoxycholic acid (*baiA-I* (black)), and (iii) hydrogen sulfide (*dsrAB* (blue)) are shown in (**a**). In (**b**), gene abundances of individual carriers are depicted with taxa affiliated with *Enterobacteriaceae*, underlined. &: affiliated with metagenomic species; Clostridium sensu. str.: *Clostridium* sensu stricto; Clostridiales bact.: SAMEA3545284 (unclassified *Clostridiales*); UBA Human: *Clostridiales bacteria* UBA6412, UBA4701, and UBA5888; Firmicutes CAG:103: *Firmicutes bacterium* CAG:103; unclass Lachnosp.: unclassified *Lachnospiraceae*

Abundances of individual pathofunctions were not associated with each other (Fig. 3a). For pathways encoding TMA formation, *cutCD* and *grdH* correlated in ten datasets, whereas *cntAB* and *cutCD* did only correlate in the four Asian-derived datasets (Fig. 3a). Finer scale analysis based on individual genera demonstrated high co-occurrence between *cntAB-* and *cutCD*-containing genera of the *Enterobacteriaceae* that were all located in one network module (Fig. 3b). Members harboring *cutCD* as well as *baiA-I* were scattered across the entire network demonstrating their distinct ecological behavior, whereas all main *grdH*-encoding taxa were connected and abundances of the two H_2_S producers did closely correlate.

Specific taxa-function analyses (see in Additional file 4) suggest that taxonomy-based diagnostic approaches can be useful to estimate the abundances of certain pathofunction-carrying groups such as the two major *dsrAB*-containing taxa *Desulfovibrio* and *Bilophila* and the *cntAB*-exhibiting *Enterobacteriacea Escherichia*/*Shigella* and *Klebsiella*, where abundances of pathofunction genes linked to those genera correlated with the overall, cumulative abundances of all members of the respective taxa. This was not the case for genera containing *cutCD-* or *baiA-I*.

DNA-based diagnostics can reveal the full pathofunctional potential, whereas expression-based techniques, metatranscriptomics and metaproteomics, as well as direct measurement of activity (e.g., measurement of metabolites), are crucial for assessing actual damage potential for the host. Metatranscriptomic results from datasets XII and XIII demonstrate frequent expression of pathofunctions, however, in fewer samples compared with metagenomes, except for *dsrAB* that was increasingly detected at the RNA level (Table 3). It should be noted that demonstrating the true absence of both genes and their expression is not possible. Given a total bacterial load of 10^12^ g^−1^ feces, the sequencing depth of metagenomics/metatranscriptomics data only provides insights into features encoded by abundant bacteria that are represented in the top orders of magnitude, and it remains elusive whether the absence of counts indicates abundance/expression below the limit of detection or true absence. Median relative expression ratios (i.e., RNA/DNA) were roughly 1 for all pathofunctions, whereas *baiA-I* (only in XII) and particularly *dsrAB* showed much higher expression as compared with DNA-based results. Expression of the Rieske-type oxygenase *CntA* was not detected in any sample indicating inactivity under strictly anaerobic conditions of the colon and suggests upper intestinal sites with oxygen availability as its main activity sites. *CntAB* results exemplify that high turnover rates of mRNA make expression-based diagnostics very sensitive, yet results based on fecal samples probably strongly bias the conclusions on activity in the upper intestinal locations. Proteins are more stable than mRNA; however, proteomics data cannot provide the same depth of information, and the detection of lowly expressed pathofunctions is hampered. For *baiA-I* and *dsrAB*, positive correlations between abundance and expression were observed, whereas for *cutCD* and *grdH*, correlations were only observed in one of the two datasets. In both datasets, the same taxa that prevailed in metagenomes displayed highest transcript levels (Fig. 2, Additional file 5). Longitudinal analyses revealed higher temporal variation in pathofunction expression than in gene abundance data, and only at the latter level stability was higher during the short time interval (1 to 3 days) compared with the results derived from the 6-month span (Additional file 5).

**Table 3:** Comparison of metagenomic and metatranscriptomic data (datasets XII and XIII)

	Dataset XII					Dataset XIII				
	MTG%	MTX%	Rho	*p*	Ratio	MTG%	MTX%	Rho	*p*	Ratio
*CutCD*	95.68*	83.33	0.09	0.42	0.72	99.67*	68.09	0.11	0.06	0.95
*CntAB*	33.45*	0.00	nd	nd	nd	14.79*	0.00	nd	nd	nd
*GrdH*	80.58*	48.72	0.27	0.02	0.80	94.06*	31.35	0.09	0.14	0.75
*BaiA-I*	65.11*	28.21	0.22	0.05	3.15	97.69*	62.38	0.22	< 0.01	1.20
*DsrAB*	55.40	70.52*	0.50	< 0.01	3.92	87.78	92.76*	0.31	< 0.01	20.7

The percentage of samples harboring individual pathofunctions in metagenomes (MTG%, *n* = 278 (XII); *n* = 311 (XIII)) and metatranscriptomes (MTX%, *n* = 78 (XII); *n* = 305 (XIII)) as well as correlations between abundance and expression levels (Spearman’s rho and *p* value) based on matched MTG/MTX samples (*n* = 78 (XII); *n* = 304 (XIII)) are shown. Ratio refers to the median RNA/DNA results of matched samples where only pairs that showed values > 0 at both levels were considered. nd: not determined. Seven metatranscriptomic samples and one metagenome from dataset XIII were omitted due to low sequencing depth (< 10^5^ reads). Significant differences (*p < 0.05) between abundance and expression based on generalized linear models are indicated

Targeted metabolite measurement provides another diagnostic level and has proven very useful in the discovery of pathofunctions [35] as it circumvents the need to detect the total gene pool of a given pathofunction, which can be challenging for the sequence-based omics techniques, particularly if bacterial carriers or enzymatic pathways have not yet been comprehensively identified. Metabolite measurements will remain indispensable for diagnostics and monitoring processes in the future. In conclusion, it is desirable to apply a combination of techniques targeting distinct levels to fully grasp both the pathofunctional potential and its actual activity together with resolving all individual taxa involved in order to perform accurate diagnostics.

### Implications of the pathofunction concept for risk assessment and development of intervention strategies to improve host health

**Fig. 4:**
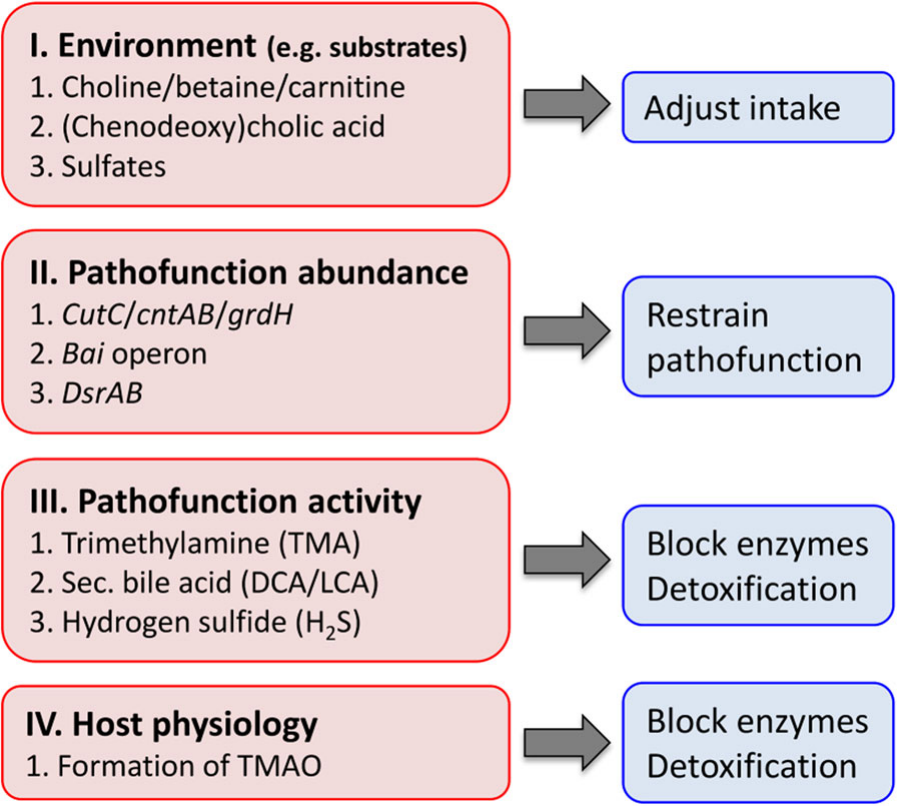
Schematic representation of the four main levels governing pathofunction activity and potential host damage (red)—details on the three examples, i.e., the formation of trimethylamine (TMA, via *CutCD*, *CntAB*, and *GrdH*), the secondary bile acids lithocholic/deoxycholic acid (LCA/DCA, via enzymes encoded in the *bai* operon), and hydrogen sulfide (H_2_S, via *DsrAB*) are given. Possibilities for intervention are shown in blue. For more information see text

Subject-specific risk assessment and development of appropriate intervention strategies requires a basic understanding of pathofunctions and respective bacterial carriers including their interaction with the surrounding microbiota and the host. In Fig. 4, we defined four broad levels that can provide a guideline for exposing individuals at high risk and for designing interventions to restrain pathofunction activity. In brief, the first level describes environmental conditions for potential pathofunction activity, primarily availability of (dietary) precursor substrates, whereas the second level represents abundances of pathofunctions and assemblages of carrier communities. Only the interplay of both levels leads to pathofunction activity that potentially causes damage to the host, which is denoted by the third level. Finally, host physiology can also be crucial for assessing actual damage risk and has to be considered as well (level 4).

The schema provides a basic guideline for risk assessment that requires adjustments for each pathofunction considering individual features. For instance, presence of TMA and LCA/DCA producers (level 2) does not imply availability of precursor substrates (level 1), because alternative energy/carbon sources are usually available for their growth. In other words, the detection of specific pathofunctions represents a minor risk for host damage unless respective substrates are available as well. This is supported by the metatranscriptomic data where transcripts were detected in fewer samples compared with gene abundance results, and only *baiA-I* showed a positive correlation between gene abundance and expression (Table 3). In contrast, reduction of sulfur compounds is the main energy conservation process for sulfate-reducing bacteria, and increased abundances are most probably directly coupled to the elevated production of H_2_S as indicated by gene expression results that correlated with gene abundance data (Table 3). Per definition, enzymes that catalyze the formation of precursors of harmful metabolites such as choline from phosphatidylcholine or sulfate from host mucus are not pathofunctions as their products do not harm the host; however, they can play an important role and may be considered for risk assessment. In case of TMA and secondary bile acids, substrates are usually available at low amounts, yet scenarios providing high precursor supplies such as choline/carnitine rich diets or high fat intake (promoting secretion of bile) are frequently occurring. Thus, diet is a key element, and comprehensive measures on both dietary components and community functions are needed to establish specific links between intake of precursing substrates, abundances, and expression of particular pathofunctions and risk for host damage. However, in practice, considering general nutritional habits for risk assessment might often be more useful. For instance, diets high in protein can promote the formation of various detrimental putrefaction products (if bacteria carrying respective functions are present) [36], and it makes little sense trying to single out each amino acid (with respective pathofunction(s)) as separate risk factors, because interventions focusing on the reduction of specific amino acids are impracticable. Rather, the overall protein intake could be lowered in individuals that harbor bacteria carrying pathofunctional-specific putrefaction pathways at elevated concentrations in order to attenuate the risk of host damage.

Host physiology can play a crucial role as well where, similar to opportunistic pathogens, certain pathofunctions are only harmful in susceptible hosts, which is exemplified by the formation of branched-chain amino acids that are proposed to contribute to insulin resistance only in obese subjects [37]. Also for TMAO-specific risk assessment, host physiology might be included. Both genetic defects, namely, trimethylaminuria, where FMO activities are absent, and genotypic (gender) differences in the potential to form TMAO, with higher enzyme activities in women compared to men, were described [38, 39].

Treatment can act on any of the outlined levels, yet broad, multilevel interventions such as limiting intake of precursors together with the reduction of nutritional niches of carriers, accompanied by boosting detoxification mechanisms, are probably most successful. Targeting nutrition (level 1) is attractive as it interferes at the initial stages reducing pathofunction activity. Furthermore, dietary precursors provide a common therapeutic target independent of the composition of bacterial carriers. Precision interventions become more difficult if (i) multiple, universal precursors are involved (e.g., formation of ammonia); (ii) substrates are essential for host health (e.g., choline); are (iii) of endogenous origin (primary bile acids); or (iv) do not involve any precursors (e.g., bacterial proteases). As discussed above, broader dietary interventions might often be more realistic. An example provides patients suffering from trimethylaminuria (accumulation of TMA in body fluids), who are advised to avoid specific foods like red meat and eggs in order to limit the intake of dietary precursors for the formation of TMA [38].

Restraining abundances of pathofunctions and growth of carriers (level 2) can be another intervention goal. Use of antibiotics is only advisable in severe cases, and rather gentle, more focused interventions are desirable, where overall community compositions are not fundamentally altered. Targeting broader groups like *Enterobacteriaceae* that are associated with several pathofunctions by reducing oxygen influx and electron acceptors for anaerobic respiration could be effective [4], whereas precision treatment specifically targeting individual carriers represents an attractive, more focused approach. However, the latter becomes particularly challenging if taxonomically diverse communities that occupy various niches in the gut ecosystem are involved. For instance, TMA-producers encompass a myriad of diverse taxa encoding distinct metabolic pathways, where carrier community assemblages can greatly differ between subjects [7]. Individualized interventions adjusted for each community type might be appropriate to narrow the spectrum of targets. Furthermore, stimulating commensals that compete for growth substrates with pathofunction carriers could be effective to restrain carriers, especially if closely related bacteria that lack pathofunctions and display large niche overlaps with carriers are involved. For several key members exhibiting choline lyase (TMA) and genes for DCA/LCA formation, phylogenetically closely related, pathofunctionally inactive strains have been isolated [7]. Administration of an array of such strains along with appropriate substrates for providing a competitive advantage over pathofunction-carrying bacteria might be applied for precision outcompeting of carriers.

Blocking activity of pathofunctions represents another target to avoid host damage (level 3). An elegant, successful therapeutic example is the application of 3,3-dimethyl-1-butanol, a structural analog of choline, which inhibits TMA lyases of gut microbiota [40]. Detoxification mechanisms by autochthonous communities provide additional, appealing targets for treatment. A prominent approach represents “Archaebiotics” that refers to the use of TMA-depleting methanogens converting TMA to DMA [41]. The recently identified iso-bile acid pathway in certain *Ruminococci* that degrades secondary bile acids LCA/DCA serves as another example demonstrating autochthonous bacteria as potential detoxifiers [42]. However, interventions at this level represent the last resort, where detoxification of harmful metabolites is directly competing with host absorption, and detailed information on detoxification kinetics will be crucial to assess applicability for treatment. Finally, altering host physiology to attenuate pathofunction virulence (e.g., reducing TMAO formation in the liver) or to promote detoxification mechanisms such as increasing the capacity of colonic epithelial cells to oxidize H_2_S [18] might represent additional intervention targets.

## Conclusions

The opportunity of modulating gut microbiota to promote host health is increasingly recognized, yet mechanisms underlying host-microbiota interactions are still poorly understood and targets for treatment remain largely elusive. Here, we focused on the concept of pathogenic functions of gut microbiota that play a role in non-communicable disorders and provide a guideline that can assist their diagnostics, risk assessment, and the development of treatment strategies. Insights into features of human microbiota damaging the host are in its infancy and the pathofunctional spectrum is largely unexplored. The discovery of new pathofunctions can pose major challenges as a manifestation of the disease often requires long-term exposure, which complicates appropriate experiments using model systems. Nevertheless, Koch’s postulates can be applied to establish a particular function as pathogenic, when initiating or increasing its activity in a suitable host causes disease as convincingly demonstrated for TMA(O) in a mouse model [8]. However, even the three metabolites investigated in this study are not exclusively regarded as being harmful. For instance, DCA plays a role in colonization resistance against *Clostridioides difficile* [43], and moderate levels of H_2_S were ascribed beneficial effects [18] exemplifying the need for establishing dose-dependent information for accurate risk assessment where host damage might not correlate with pathofunction activity in a linear fashion. Cohort studies applying longitudinal sampling together with technological advancements including multiomics technologies provide encouraging environments for revealing additional pathofunction candidates.

For diagnostics, comprehensive databases encompassing the full taxonomic and biochemical diversity play a central role, and adjusted workflows to capture low abundant features might be required, which explains the limited results related to the three pathofunctions obtained in original studies (Additional file 1). Often, key pathofunction carriers are unknown, even for those presented in this study. Metagenomic species identified based on genome reconstructions from metagenomic data circumvent the need for cultivation and proved useful in this study where they served as key references (Fig. 2, Additional file 3). It is possible to estimate their intestinal niches based on genomic features; however, the ecological understanding of such bacteria will be limited due to inability to perform defined experiments. The need to isolate and cultivate key pathofunction carriers remains eminent.

Complete eradication or blocking of all pathofunctions in a given community is difficult, and rather restraining pathofunction abundance and activity will be in focus in the future. Major tasks will involve quantitative monitoring of long-term exposure dynamics to establish concentration thresholds for risk assessment and for defining successful treatment. Although the so-called “healthy microbiota”, derived from symptom-free subjects, provides a first reference, it is an imperfect benchmark that is vaguely defined and contains a myriad of pathofunctions. In our opinion, reducing pathofunctions will improve host well-being, even in the healthy population, and particularly bears great potential when it comes to increase our lifespan and to promote healthy aging where chronic disorders play a central role.

## Methods

### Databases

#### *CutCD*, *cntAB*, and *grdH* (TMA formation)

References for *cutCD* and *cntAB* provided in [7] were updated (PATRIC genomes, *n* = 107,042, June 2017). To identify genes encoding the β-subunit of betaine reductases (*grdH*), the same genomes were screened (*hmmsearch*, HMMER 3.1b1, hmmer.org) using a hidden Markov model (HMM) constructed from the following protein references based on [12]: 742765.5.peg.3571, 1133568.3.peg.2056, 1125712.3.peg.1676, 999407.4.peg.5417, 1531.8.peg.5368, 712357.3.peg.735, 552395.3.peg.1966, 411465.10.peg.881, 457415.3.peg.2639; sequences were trimmed from the 3-prime end till selenocystein as this part was often lacking in PATRIC sequences. A phylogenetic tree was constructed (FastTree (v. 2.1.8) [44] using the JTT+CAT model) from all sequences that displayed HMM scores > 100 and ≥ 80% coverage to the model, and distances between the branch tips and the top-scoring sequence were determined using cophenetic.phylo function in R (v. 3.1.2) (package: ape, v. 3.4). A steep HMM score drop was obvious at around 550 that correlated with the increases in phylogenetic distances, and all sequences displaying a score > 500 were considered as true *grdH* yielding 346 candidates (Additional file 6A). Selected sequences form a clade in the tree separated from sequences encoding distinct functionality (Additional file 6B) in the selenoproteins of the glycine/betaine/sarcosine/d-proline reductase family.

#### *BaiA-I* (LCA/DCA formation)

Full-length HMM models were constructed for *bai A-I* genes using sequences based on Reference [15] and manual BLAST searches (PATRIC genome IDs: 1505.29, 1505.7, 1232454.3, 500633.7, 553973.6, 411468.9, 658665.3, 658085.3, 1123009.3). All PATRIC genomes were screened, and cutoffs were set after obvious HMM score drops for each gene. Subsequently, all genomes exhibiting ≥ 4 genes in synteny (defined as being separated by ≤ 10 genes based on locus tag) were selected as candidates. Additional manual inspections on NCBI yielded *baiA,E,F* for genomes 165185.6 and 165186.4 that exhibited only three genes in initial searches. For verification, phylogenetic trees were constructed for all genes (*baiA-I*) where sequences considered as true *bai* formed a clade separate from lower-scoring genes that were not considered encoding the functions of interest. Finally, 60 bacteria exhibiting the *bai* operon were revealed (46 were *Clostridium sordellii* strains).

#### *DsrAB* (H_2_S formation)

In the database provided by Müller et al. [16], subunits A and B were split and all sequences displaying > 70% length to the references from *Desulfovibrio vulgaris* (NC_002937) were subjected to FrameBot analysis (v. 1.2, in default mode [45], with HMMs derived from FunGene [46]); all protein sequences were subsequently used in BLAST searches (see below).

C*utCD* (*n* = 1) and *bai* (*n* = 6) genes derived from metagenomic species available in PATRIC and from reference [47] were added to the databases (only those found in feces and displaying protein sequence similarities > 70% and < 95% to references were considered). Taxonomic affiliations were based on the RDP taxonomy where 16S rRNA gene sequences of genomes were retrieved and subjected to classification using the RDP classifier [48] as described previously [7].

### Screening for pathofunctions in metagenomic/transcriptomic datasets

Raw reads of all samples were downloaded from the European Nucleotide Archive (http://www.ebi.ac.uk/ena) and the Sequence Read Archive (https://www.ncbi.nlm.nih.gov/sra), quality filtered for an average *Q* score ≥ 20 and length ≥ 70 using Trimmomatic [49]. Filtered reads were BLASTED (blastx using DIAMOND [50]) against databases described above, and the top-hitting reference was recorded if the query alignment was ≥ 20 amino acids showing ≥ 70% similarity to references. Three single-copy housekeeping genes encoding 50S ribosomal protein L2 (*rplB*), recombinase A (*recA*), and CTP-synthase (*pyrG*) from all PATRIC genomes were included in BLAST searches [51]. For *cutCD*, *cntAB*, *grdH*, and *baiA-I*, sequences below the set HMM threshold were included at this stage (as done in [7]) to avoid the possibility of false-positive counts derived from those related genes. Matching read counts were gene length corrected using the median length of respective reference sequences. For each sample, median counts associated with individual pathofunctions were used to calculate pathofunction abundances relative to mean counts linked to the three housekeeping genes of all PATRIC genomes (representing total genomes in a sample) as performed previously [51]. All genes of a pathway had to be detected for considering a pathway being present (for *baiA-I*, the cutoff was set at four genes). For TMA, calculations were performed for each pathway separately. Thus, throughout the manuscript, pathofunction “abundance” refers to the percentage of bacteria carrying that function. Metatranscriptomic data are presented relative to the mean expression of the three housekeeping genes. Taxa abundance (and expression levels, respectively) comprising individual pathofunctions are shown on the genus level calculated from the cumulative count data of all genes derived from the same genus in pathofunction reference databases relative to mean counts of the three housekeeping genes of all PATRIC genomes. For taxa not affiliated with a genus such as unclassified *Clostridiales bacterium* SAMEA3545284 or *Firmicutes bacterium* CAG:103, strain names are given.

Statistical analyses were performed in R: Spearman correlation (package *Hmisc*), *q* values (package *fdrtool*), Student’s *t* test (function *t.test*), logistic regression (function *glm*) (family = binomial), and area under the receiver-operating characteristic curve (package *pROC*). Generalized linear mixed-effects models were constructed for each disease (function *glmer* (family = binomial) from package *lme4*) using dataset as a random effect. Differences in abundance and expression of pathofunctions were assessed based on generalized linear models (function *glm* (family = binomial)) using presence/absence data and total counts as offset in order to adjust for lower sequencing depth in metatranscriptomic data (3.97 × 10^6^ ± 1.44 × 10^5^ vs. 2.62 × 10^6^ ± 2.47 × 10^5^ (XII) and 3.74 × 10^6^ ± 8.40 × 10^4^ vs. 2.99 × 10^6^ ± 1.39 × 10^5^ (XIII) (mean ± SE)). FDR-corrected Mann-Whitney *U* tests were done in QIIME (v. 1.9.1, [52]). Violin plots and heatmaps (based on log-transformed, abundance data (log(*x* + 1)) were constructed in R using the packages gplots (v. 2.17.0) and ggplot2 (v. 2.2.1). Networks were visualized in cytoscape (v. 2.3.1, http://cytoscape.org, preferred layout with some modifications) considering correlations (*p* and *q* < 0.05, Spearman’s rho ≥ 0.35) that were detected in at least three datasets (*n* = 12).

## Additional files

Additional file 1: Indications in original studies for differential abundance of the three pathofunctions, namely, the formation of trimethylamine (TMA), secondary bile acids lithocholic/deoxycholic acid (LCA/DCA) and hydrogen sulfide (H_2_S) between patients and respective controls based on functional and taxonomy-based analyses. Data was queried for functions based on function names and KEGG Orthologies (provided for most studies. *CutC/D*: K20038/K20038, *cntA/B:* K22443/ K22444, *grdH:* K21579, *baiCD/E:* K15870/K15872, *dsrA/B:* K11180/ K11180). Taxonomic data was screened for key taxa that correlated with function based on information given in Additional file 4 (TMA (*cntAB*): *Escherichia/Klebsiella*, TMA (*grdH*): *Dorea*, H_2_S: *Bilophila/Desulfovibrio*). *Clostridium scindens* (DCA/LCA) was included as well. Jie et al. [21] (dataset I) additionally analyzed datasets III, VI and X for TMA associated genes based on selected reference sequences and results are also displayed. Forslund et al. [24] included studies III-V into their analysis and reported a trended increase (*p* = 0.07) for MF0100 (dissimilatory sulfate reduction) in T2D patients across studies. ?: no information on that function/taxa was retrieved, =: no difference between patients and control group, CD: Crohn’s disease, CRC: colorectal cancer, CVD: cardiovascular disease, HGC: high gene count group, MLG: metagenomic linkage group, T2D: type 2 diabetes, UC: ulcerative colitis. (PDF 56 kb)

Additional file 2: Area under the receiver-operating characteristic curve (AUC) applying generalized linear mixed-effects models for each disease based on abundances of genes encoding three pathofunctions, namely, the formation of (i) trimethylamine (TMA: *cutCD*, *cntAB* and *grdH*), (ii) the secondary bile acids lithocholic/deoxycholic acid (*bai* operon) and (iii) hydrogen sulfide (dsr genes) are displayed as well as results from combined data (All). AUCs of individual datasets and of diseases represented by only one dataset based on logistic regression are indicated after arrows on the right and at the bottom, respectively. Data encompassed patients suffering from cardiovascular disease (CVD: I, II), type 2 diabetes (T2D: III-V), obesity (Obese: VI), colorectal cancer (CRC: VII, VIII, IX), liver cirrhosis (Cirr: X) and inflammatory bowel disease (IBD: ulcerative colitis (UC) and Crohn’s disease (CD), XI and XII). Samples from type 1 diabetes (dataset IV), adenomas (datasets VII and VIII) and CD of dataset XI were not considered. For details on individual datasets see Table 2. (PDF 2400 kb)

Additional file 3: Neighbor joining tree of *baiCD* sequences. Sequences from *C*. *sordellii* were merged. On the right, taxonomic affiliations (on genus level) are given with amount of reads (as % of total *bai* associated reads) from omics data (*n* = 2975) linked to individual taxa. &: Sequences derive from metagenomic species (including information on isolation source (host)). (PDF 58 kb)

Additional file 4: The number of genomes of major genera exhibiting individual pathofunctions is displayed, where the percentage of bacteria exhibiting the pathofunction of all members of that genus is given in brackets (Number Genomes (% of genus)). Correlations between pathofunction abundances affiliated with a genus and abundances of all members of respective genera (based on three house-keeping genes) are shown as well (CorrelationTaxonomy). The number of datasets displaying a correlation (*p* and *q* < 0.05 and Spearman’s rho > 0.35, *n* = 12) is given and Spearman’s rho (average ± standard deviation) is displayed in brackets. Datasets IV and VI were merged as they derived from one source and have overlapping samples in the healthy control groups. ND: not determined. (PDF 48 kb)

Additional file 5: Metagenomic and metatranscriptomic analyses of genes encoding three pathofunctions, namely, the formation of (i) trimethylamine (*cutCD*, *cntAB*, *grdH*), (ii) the secondary bile acids lithocholic/deoxycholic acid (*baiA-I*) and (iii) hydrogen sulfide (*dsrAB*) in 78 healthy male adults sampled at four time points [53]. Top panels display pathofunction abundance (A) and expression (B) considering all samples. Results of individual taxa are shown in the panels below (C, D), where relative abundances, i.e., percentage of each taxon from total pathofunction abundance/expression data, are shown. Panels E and F display temporal variability of both levels where $ indicates significant higher variability (*p* < 0.05) in gene expression compared with gene-abundance results and *,+ indicate increased (*p* < 0.05, *p* < 0.1) variability in the six months interval compared with the short time intervals (1 to 3 days; Student’s *t* test). Temporal variability was calculated as abundance/expression differences between two time-points relative to the higher value ranging from 0% (no change) to 100% (absent at one time-point). §: Affiliated with *Enterobacteriaceae*, &: affiliated with metagenomic species, Clostridium sens. str.: *Clostridium* sensu stricto, Clostridiales bact.: SAMEA3545284 (unclassified *Clostridiales*), unclass Lachnosp.: unclassified *Lachnospiraceae*. Seven metatranscriptomic samples and one metagenome were omitted due to low sequencing depth (< 10^5^). (PDF 1185 kb)

Additional file 6: Results of screening procedures for *grdH* sequences. In Panel A obtained unique proteins are depicted along the x-axis, sorted according to their similarity to the constructed hidden Markov chain model (HMM) represented by the primary y-axis. The secondary y-axis shows phylogenetic distances to the top-scoring sequence (triangles). Sequences considered as true *grdH* are shown in blue. Below (B) a tree of all sequences from Panel A is displayed with the clade containing true *grdH* sequences highlighted in blue. Sequences encoding glycine reductase (*grdB*: Q9R4G8-1) and sarcosine reductase (*grdF*: O86186-1) are shown in pink (UniProt IDs are given). (PDF 127 kb)
