# Supplementary material for: Pathogenic functions of host microbiota
Source: Microbiome. 2018 Sep 28;6:174. doi: 10.1186/s40168-018-0542-0 (PMC6162913; doi:10.1186/s40168-018-0542-0)
Supplement: Supplementary file 1 — Indications in original studies for differential abundance of the three pathofunctions, namely, the formation of trimethylamine (TMA), secondary bile acids lithocholic/deoxycholic acid (LCA/DCA) and hydrogen sulfide (H2S) between patients and respective controls based on functional and taxonomy-based analyses. Data was queried for functions based on function names and KEGG Orthologies (provided for most studies. CutC/D: K20038/K20038, cntA/B: K22443/ K22444, grdH: K21579, baiCD/E: K15870/K15872, dsrA/B: K11180/ K11180). Taxonomic data was screened for key taxa that correlated with function based on information given in Additional file 4 (TMA (cntAB): Escherichia/Klebsiella, TMA (grdH): Dorea, H2S: Bilophila/Desulfovibrio). Clostridium scindens (DCA/LCA) was included as well. Jie et al. [21] (dataset I) additionally analyzed datasets III, VI and X for TMA associated genes based on selected reference sequences and results are also displayed. Forslund et al. [24] included studies III-V into their analysis and reported a trended increase (p = 0.07) for MF0100 (dissimilatory sulfate reduction) in T2D patients across studies. ?: no information on that function/taxa was retrieved, =: no difference between patients and control group, CD: Crohn’s disease, CRC: colorectal cancer, CVD: cardiovascular disease, HGC: high gene count group, MLG: metagenomic linkage group, T2D: type 2 diabetes, UC: ulcerative colitis. (PDF 56 kb) [file 40168_2018_542_MOESM1_ESM.pdf]

			Function			Taxonomy		
	**Disease**	**Reference**	**TMA**	**DCA/LCA**	**H2S**	**TMA**	**DCA/LCA**	**H2S**
I	CVD	Jie *et al*, 2017	*CntAB*↑; *cutCD* =	**?**	*DsrAB*↓	*Escherichia* and *Klebsiella* MLG*s*↑	*C. scindens*↑	*Bilophila* MLG↓; *Desulfovibrio* MLG↑
II	CVD	Karlsson *et al*, 2012	Phospahtidylcholine to TMA =	**?**	**?**	**?**	**?**	**?**
III	T2D	Qin *et al*, 2012	**?**, Jie *et al: cutCD*↑; *cntAB* =	**?**	*DsrA*↑	*Escherichia* and *Klebsiella* MLGs↑	*C. scindens* MLGs↑	*Desulfovibrio* and *Bilophila* MLGs↑
IV	T2D	Forslund *et al*, 2015	**?**	**?**	MF0100 trended↑ (Ds III-V)	*Escherichia*↑;Dorea↓	*C. scindens* =	*Bilophila*↑; *Desulfovibrio* =
V	T2D	Karlsson *et al*, 2013	**?**	**?**	**?**	*Dorea* MGC↓	**?**	**?**
VI	Obesity	Le Chatelier *et al*, 2013	**?**, Jie *et al*: *cutC* =; *cutD*↓; *cntAB* =	**?**	**?**	**?**	**?**	*Desulfovibrio*↑ in HGC
VII	CRC	Feng *et al*, 2015	**?**	**?**	**?**	*E.coli*↑	**?**	*Bilophila*↑
VIII	CRC	Zeller *et al*, 2014	**?**	**?**	**?**	*Dorea*↑	*C. scindens*↑	*Bilophila* and *Desulfovibrio*↑
IX	CRC	Vogtmann *et al*, 2016	**?**	**?**	*DsrAB* =	*Escherichia* trended ↑; *Klebsiella* =	*C. scindens* =	*Bilophila* and *Desulfovibrio* =
X	Cirrhosis	Qin *et al*, 2014	**?**, Jie *et al*: *cutCD*↓; *cntAB*↓	**?**	*DsrAB*↓	*Escherichia* and *Klebsiella*↑	**?**	*Bilophila*↓
XI	UC	Qin *et al*, 2010	**?**	**?**	**?**	**?**	**?**	**?**
XII	UC/CD	Schirmer *et al*, 2018	**?**	**?**	**?**	*E. coli*↑ in CD	**?**	**?**
