# Supplementary figures and images for: Pathogenic functions of host microbiota
Source: Microbiome. 2018 Sep 28;6:174. doi: 10.1186/s40168-018-0542-0 (PMC6162913; doi:10.1186/s40168-018-0542-0)

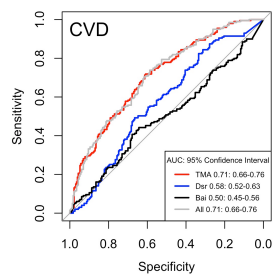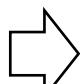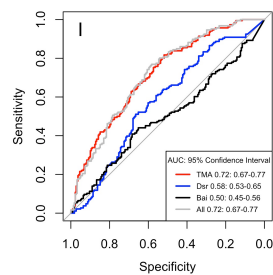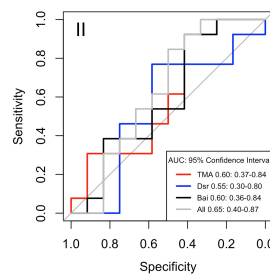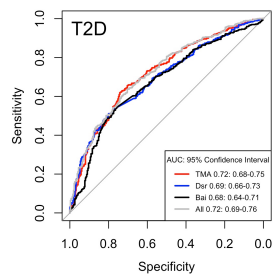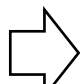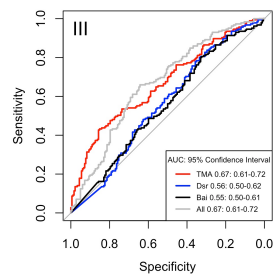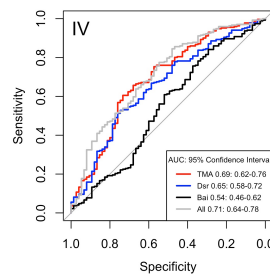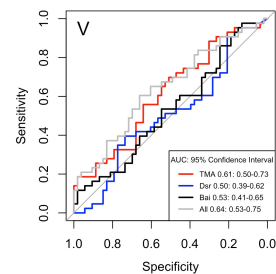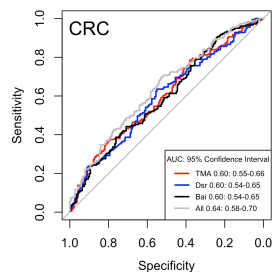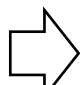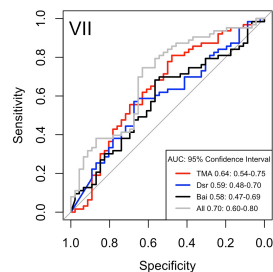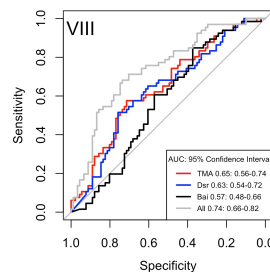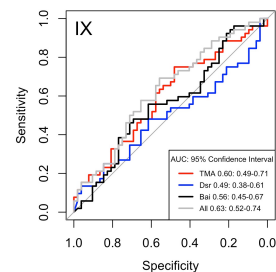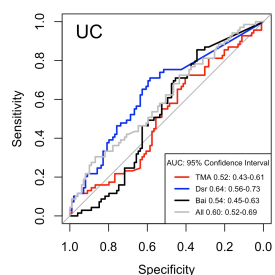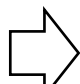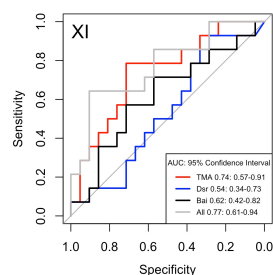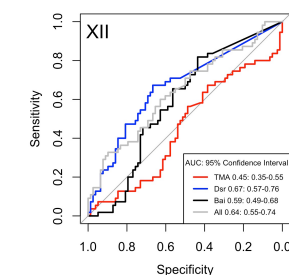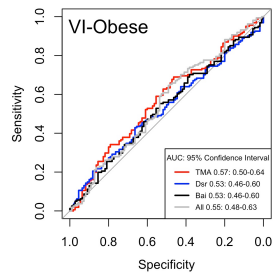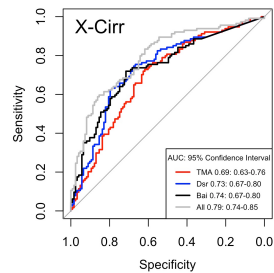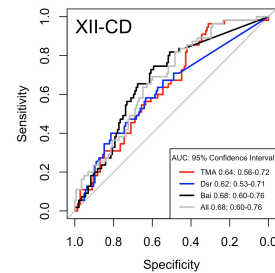

Supplement: Supplementary file 2 — Area under the receiver-operating characteristic curve (AUC) applying generalized linear mixed-effects models for each disease based on abundances of genes encoding three pathofunctions, namely, the formation of (i) trimethylamine (TMA: cutCD, cntAB and grdH), (ii) the secondary bile acids lithocholic/deoxycholic acid (bai operon) and (iii) hydrogen sulfide (dsr genes) are displayed as well as results from combined data (All). AUCs of individual datasets and of diseases represented by only one dataset based on logistic regression are indicated after arrows on the right and at the bottom, respectively. Data encompassed patients suffering from cardiovascular disease (CVD: I, II), type 2 diabetes (T2D: III-V), obesity (Obese: VI), colorectal cancer (CRC: VII, VIII, IX), liver cirrhosis (Cirr: X) and inflammatory bowel disease (IBD: ulcerative colitis (UC) and Crohn’s disease (CD), XI and XII). Samples from type 1 diabetes (dataset IV), adenomas (datasets VII and VIII) and CD of dataset XI were not considered. For details on individual datasets see Table 2. (PDF 2400 kb) [file 40168_2018_542_MOESM2_ESM.pdf]

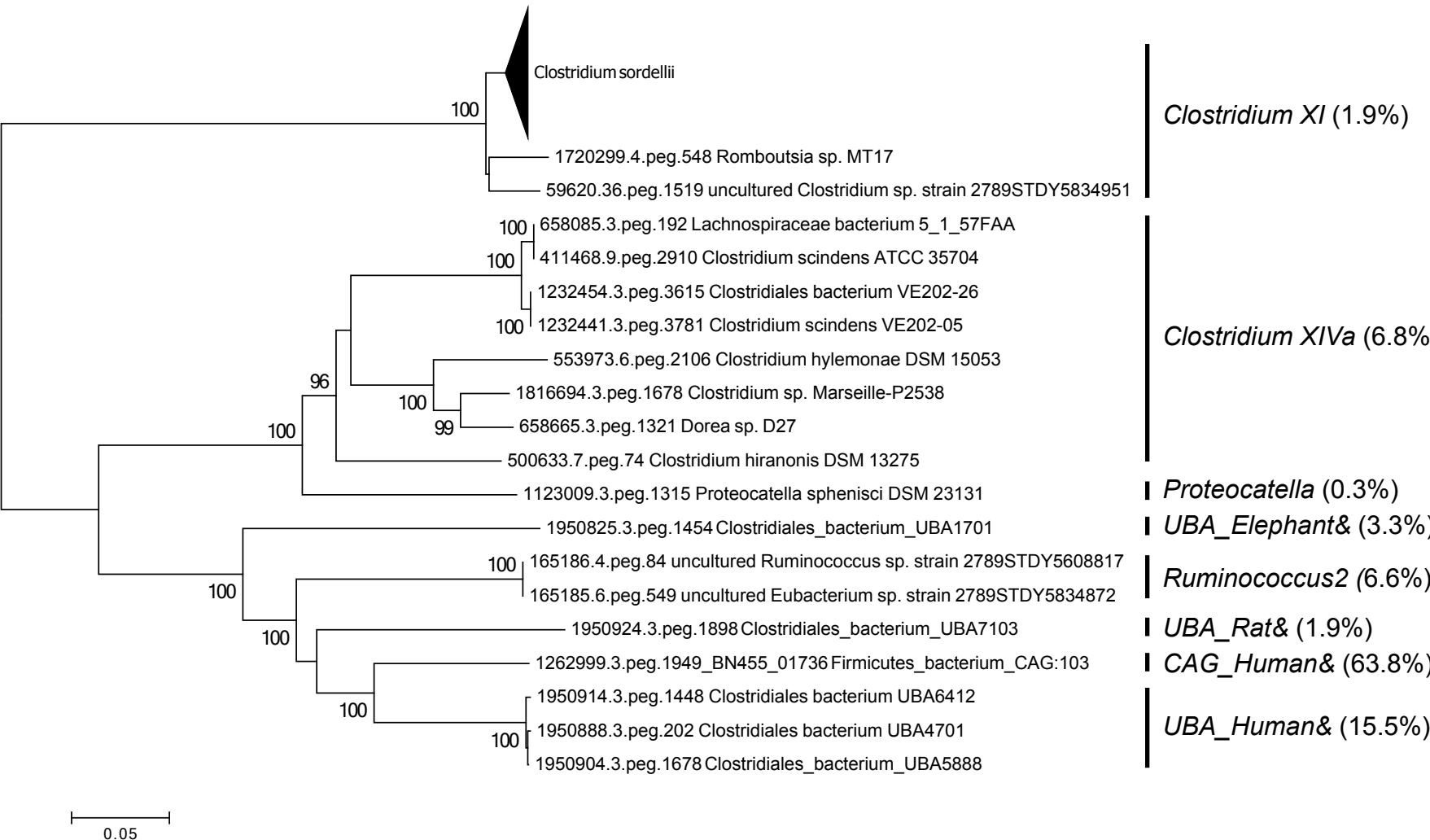

Supplement: Supplementary file 3 — Neighbor joining tree of baiCD sequences. Sequences from C. sordellii were merged. On the right, taxonomic affiliations (on genus level) are given with amount of reads (as % of total bai associated reads) from omics data (n = 2975) linked to individual taxa. &: Sequences derive from metagenomic species (including information on isolation source (host)). (PDF 58 kb) [file 40168_2018_542_MOESM3_ESM.pdf]

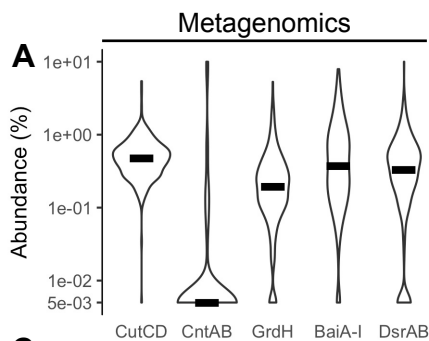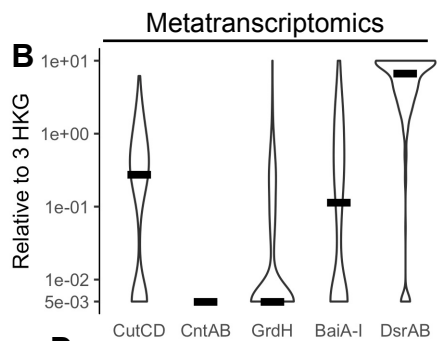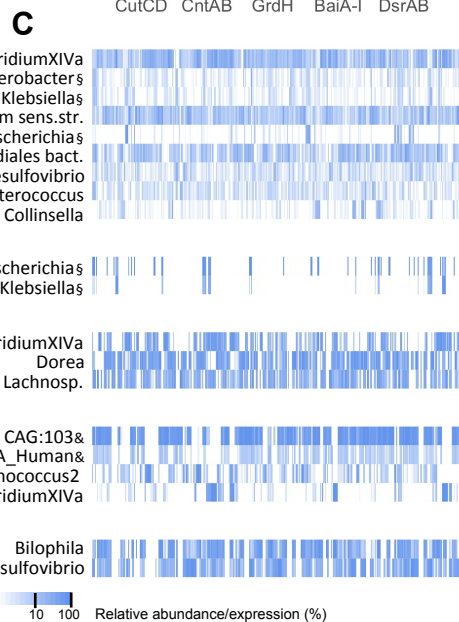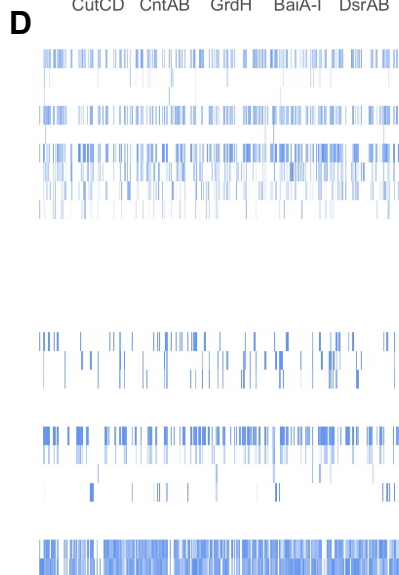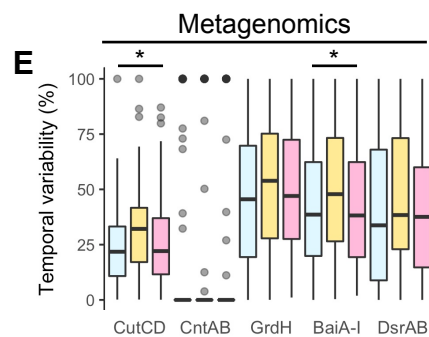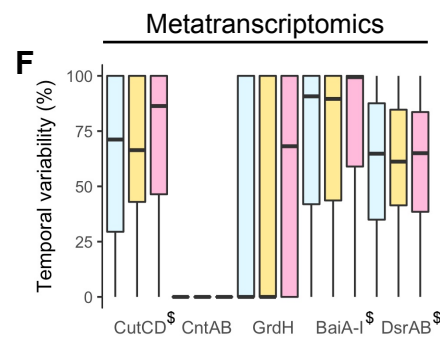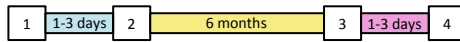

Supplement: Supplementary file 5 — Metagenomic and metatranscriptomic analyses of genes encoding three pathofunctions, namely, the formation of (i) trimethylamine (cutCD, cntAB, grdH), (ii) the secondary bile acids lithocholic/deoxycholic acid (baiA-I) and (iii) hydrogen sulfide (dsrAB) in 78 healthy male adults sampled at four time points [53]. Top panels display pathofunction abundance (A) and expression (B) considering all samples. Results of individual taxa are shown in the panels below (C, D), where relative abundances, i.e., percentage of each taxon from total pathofunction abundance/expression data, are shown. Panels E and F display temporal variability of both levels where $ indicates significant higher variability (p < 0.05) in gene expression compared with gene-abundance results and *,+ indicate increased (p < 0.05, p < 0.1) variability in the six months interval compared with the short time intervals (1 to 3 days; Student’s t test). Temporal variability was calculated as abundance/expression differences between two time-points relative to the higher value ranging from 0% (no change) to 100% (absent at one time-point). §: Affiliated with Enterobacteriaceae, &: affiliated with metagenomic species, Clostridium sens. str.: Clostridium sensu stricto, Clostridiales bact.: SAMEA3545284 (unclassified Clostridiales), unclass Lachnosp.: unclassified Lachnospiraceae. Seven metatranscriptomic samples and one metagenome were omitted due to low sequencing depth (< 105). (PDF 1185 kb) [file 40168_2018_542_MOESM5_ESM.pdf]

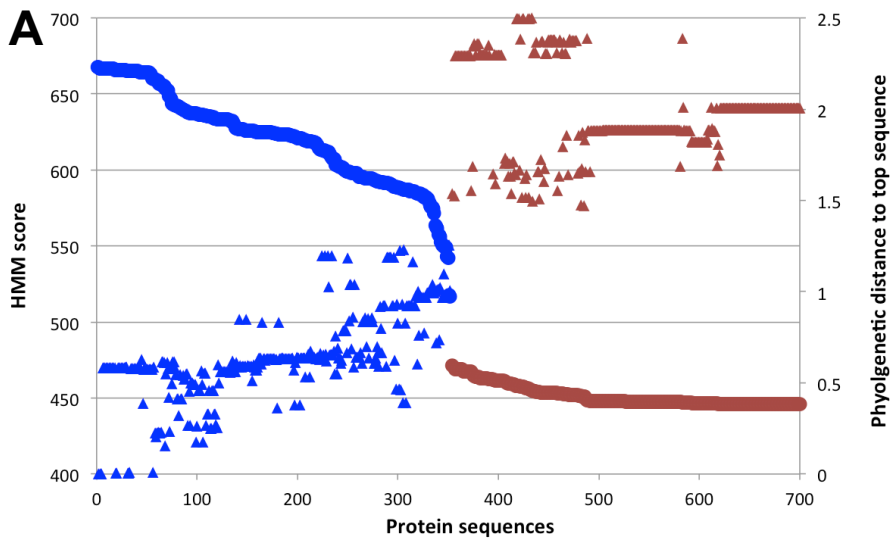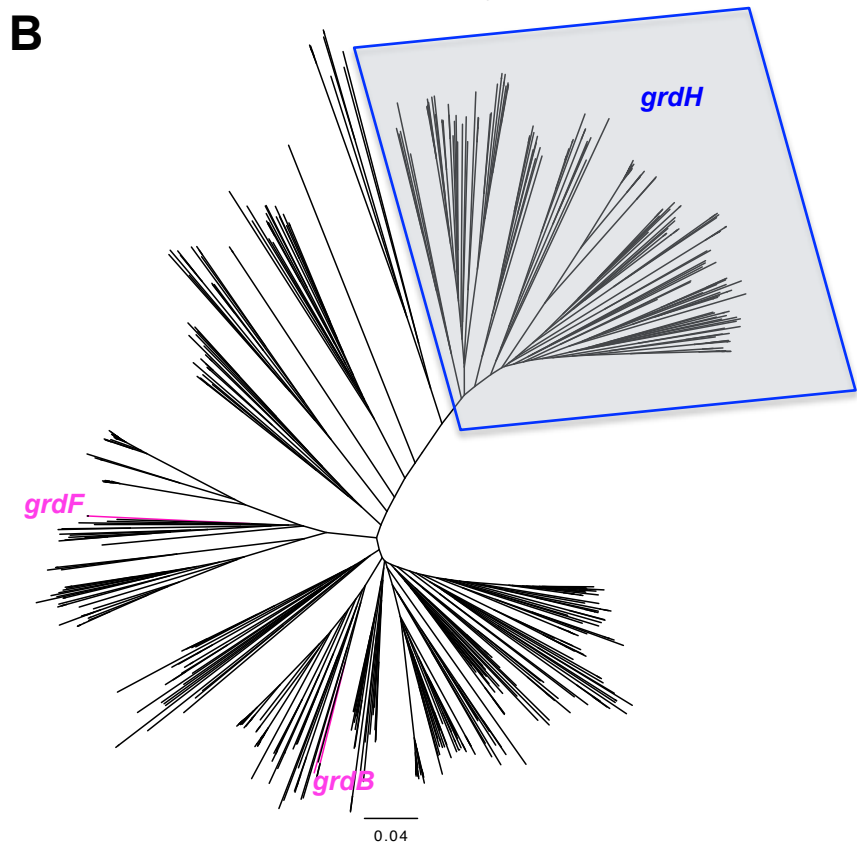

Supplement: Supplementary file 6 — Results of screening procedures for grdH sequences. In Panel A obtained unique proteins are depicted along the x-axis, sorted according to their similarity to the constructed hidden Markov chain model (HMM) represented by the primary y-axis. The secondary y-axis shows phylogenetic distances to the top-scoring sequence (triangles). Sequences considered as true grdH are shown in blue. Below (B) a tree of all sequences from Panel A is displayed with the clade containing true grdH sequences highlighted in blue. Sequences encoding glycine reductase (grdB: Q9R4G8-1) and sarcosine reductase (grdF: O86186-1) are shown in pink (UniProt IDs are given). (PDF 127 kb) [file 40168_2018_542_MOESM6_ESM.pdf]
