# Supplementary material for: Pathogenic functions of host microbiota
Source: Microbiome. 2018 Sep 28;6:174. doi: 10.1186/s40168-018-0542-0 (PMC6162913; doi:10.1186/s40168-018-0542-0)
Supplement: Supplementary file 4 — The number of genomes of major genera exhibiting individual pathofunctions is displayed, where the percentage of bacteria exhibiting the pathofunction of all members of that genus is given in brackets (Number Genomes (% of genus)). Correlations between pathofunction abundances affiliated with a genus and abundances of all members of respective genera (based on three house-keeping genes) are shown as well (CorrelationTaxonomy). The number of datasets displaying a correlation (p and q < 0.05 and Spearman’s rho > 0.35, n = 12) is given and Spearman’s rho (average ± standard deviation) is displayed in brackets. Datasets IV and VI were merged as they derived from one source and have overlapping samples in the healthy control groups. ND: not determined. (PDF 48 kb) [file 40168_2018_542_MOESM4_ESM.pdf]

| *CutCD* | Number genomes (% of genus) | CorrelationTaxonomy |
|---|---|---|
| *Clostridium XlVa* | 29 (21.80%) | 6 (0.49±0.10) |
| *Enterobacter* | 160 (30.39%) | 7 (0.56±0.11) |
| *Klebsiella* | 262 (10.99%) | 4 (0.55±0.14) |
| *Clostridium sensu stricto* | 246 (49.90%) | - |
| *Escherichia* | 232 (3.22%) | 5 (0.43±0.07) |
| *Desulfovibrio* | 16 (25.00%) | 6 (0.47±0.10) |
| *Enterococcus* | 41 (2.49%) | 2 (0.36/0.36) |
| *Collinsella* | 3 (11.54%) | 2 (0.36/0.36) |
| ***CntAB*** | | |
| *Escherichia* | 4788 (66.38%) | 10 (0.73±0.17) |
| *Klebsiella* | 1682 (70.52%) | 12 (0.64±0.18) |
| ***GrdH*** | | |
| *Clostridium XlVa* | 40 (30.30%) | 6 (0.48±0.09) |
| *Dorea* | 2 (22.22%) | 11 (0.49±0.11) |
| ***BaiA-I*** | | |
| *Clostridium XlVa* | 7 (5.30%) | 1 (0.35) |
| *Ruminococcus2* | 2 (11.76%) | 2 (0.59/0.40) |
| ***DsrAB*** | | |
| *Bilophila* | ND | 12 (0.91±0.06) |
| *Desulfovibrio* | ND | 11 (0.63±0.13) |
